# Inferring Drug–Gene Relationships in Cancer Using Literature-Augmented Large Language Models

**DOI:** 10.1158/2767-9764.CRC-25-0030

**Published:** 2025-04-28

**Authors:** Ying-Ju Lai, Li-Ju Wang, Tyler M. Yasaka, Yuna Shin, Michael Ning, Yanhao Tan, Chien-Hung Shih, Yibing Guo, Po-Yuan Chen, Hugh Galloway, Zhentao Liu, Arun Das, George C. Tseng, Satdarshan P. Monga, Yufei Huang, Yu-Chiao Chiu

**Affiliations:** 1 UPMC Hillman Cancer Center, University of Pittsburgh School of Medicine, Pittsburgh, Pennsylvania.; 2 Department of Biostatistics and Health Data Science, School of Public Health, University of Pittsburgh, Pittsburgh, Pennsylvania.; 3 Medical Scientist Training Program, University of Pittsburgh School of Medicine, Pittsburgh, Pennsylvania.; 4 Department of Electrical and Computer Engineering, Carnegie Mellon University, Pittsburgh, Pennsylvania.; 5 Department of Computer Science, The University of Texas at Austin, Austin, Texas.; 6 Department of Medicine, University of Pittsburgh School of Medicine, Pittsburgh, Pennsylvania.; 7 Department of Human Genetics, School of Public Health, University of Pittsburgh, Pittsburgh, Pennsylvania.; 8 Institute of Bioinformatics and Systems Biology, National Yang Ming Chiao Tung University, Hsinchu, Taiwan.; 9 Department of Electrical and Computer Engineering, Swanson School of Engineering, University of Pittsburgh, Pittsburgh, Pennsylvania.; 10 Department of Pharmacology & Chemical Biology, University of Pittsburgh School of Medicine, Pittsburgh, Pennsylvania.; 11 Pittsburgh Liver Research Center, University of Pittsburgh Medical Center and University of Pittsburgh School of Medicine, Pittsburgh, Pennsylvania.; 12 Organ Pathobiology and Therapeutics Institute, University of Pittsburgh Medical Center and University of Pittsburgh School of Medicine, Pittsburgh, Pennsylvania.; 13 Department of Pharmaceutical Sciences, University of Pittsburgh School of Medicine, Pittsburgh, Pennsylvania.; 14 Department of Computational and Systems Biology, University of Pittsburgh School of Medicine, Pittsburgh, Pennsylvania.

## Abstract

Understanding drug–gene relationships is essential for advancing targeted cancer therapies and drug repurposing strategies. However, the vast volume of biomedical literature poses significant challenges in efficiently extracting relevant insights. In this study, we developed an automated pipeline that leverages retrieval-augmented large language models (LLM) to infer drug–gene interactions using the most up-to-date biomedical literature. By integrating PubMed and state-of-the-art LLMs, our pipeline generates accurate, evidence-based inferences while addressing the limitations of static LLMs, such as outdated knowledge and the risk of producing misleading results. We systematically validated the pipeline’s performance using curated databases and demonstrated its ability to accurately identify both well-established and emerging drug targets. Using our pipeline, we constructed a pan-cancer drug–gene interaction network among hundreds of FDA-approved drugs and key oncogenes. In a case study on liver cancer, we identified and validated an association between *CTNNB1* mutations and enhanced sensitivity to sorafenib, highlighting a potential therapeutic strategy for this challenging mutation. To facilitate broad accessibility, we developed GeneRxGPT, a user-friendly web application that enables cancer researchers to utilize the pipeline without programming expertise or extensive computational resources. It provides intuitive modules for drug–gene inference and network visualization, streamlining the exploration and interpretation of drug–gene relationships. We anticipate that GeneRxGPT will empower researchers to accelerate drug discovery and development, making it a valuable resource for the cancer research community.

**Significance:**

This study presents a novel approach that integrates LLMs with real-time biomedical literature to uncover drug–gene relationships, transforming how cancer researchers identify therapeutic targets, repurpose drugs, and interpret complex molecular interactions. GeneRxGPT, our user-friendly tool, enables researchers to leverage this approach without requiring computational expertise.

## Introduction

Understanding the intricate relationships between genes and drugs is critical for advancing cancer treatment and improving patient outcomes. This knowledge underpins drug development by helping researchers decipher the molecular effects of drug perturbations, develop drug repurposing strategies, and optimize therapeutic efficacy (1). Drug–gene target relationships can involve direct drug binding into a gene product, such as a protein, or indirect modulation, in which genes influence drug sensitivity or drugs affect gene activity through downstream signaling pathways or other mechanisms (2, 3). Among the more than 38 million biomedical articles indexed in PubMed, many provide valuable insights into drug–gene interactions (4, 5). However, the vast volume of studies makes it challenging for researchers to stay updated and synthesize findings efficiently (6). Therefore, developing an automated pipeline to analyze literature and extract drug–gene relationships is crucial for identifying novel targets and accelerating cancer therapy discovery (7).

In recent years, large language models (LLM), such as GPT-4o developed by OpenAI, have demonstrated significant advancements in natural language processing (arXiv 2024.03.04.2303.08774). These models can perform complex biomedical tasks beyond traditional methods, such as information summarization, knowledge discovery, and complex reasoning (8–10). However, LLMs have notable limitations, such as difficulty incorporating the latest research dynamically and a tendency to generate “hallucinations,” which are plausible but misleading responses not grounded in reliable knowledge (11, 12). To address these issues, “retrieval-augmented generation” enhances LLM performance by integrating relevant external sources, such as PubMed literature (13, 14). By incorporating up-to-date data, retrieval-based models can significantly reduce hallucinations and provide more reliable, evidence-based responses (12, 15). However, as implementing these techniques requires programmatic access to databases like PubMed and advanced programming skills, a more accessible tool is needed to democratize LLMs and retrieval-augmented methods, particularly for cancer research, a rapidly evolving field.

In this study, we developed an efficient approach for inferring drug–gene target relationships in cancer using LLMs grounded in up-to-date biomedical literature and assessed its reliability. We created an automated pipeline that integrates PubMed’s Application Programming Interface (API) with LLMs to retrieve literature, summarize information, and generate inferences. This pipeline was systematically evaluated using curated drug–gene target databases, focusing on the impact of PubMed retrieval, comparisons among open-source and proprietary LLMs, and output consistency. We further applied our tool to construct a pan-cancer drug–gene interaction network covering hundreds of oncogenes and FDA-approved drugs. As a case study, we investigated liver cancer, a disease with significant treatment challenges (16, 17), by examining the traditionally undruggable *CTNNB1* mutation. We validated its role in modulating sorafenib response using data from a clinical trial and *in vitro* screens. To enhance accessibility, we developed GeneRxGPT, a user-friendly web tool implemented with the R Shiny app framework, available at https://shiny.crc.pitt.edu/generxgpt/. Given a gene, drug, and cancer type, this tool summarizes relevant PubMed literature, infers drug–gene relationship, and provides references with direct links. In summary, this study presents an efficient and accurate method for identifying drug–gene relationships, which has the potential to accelerate pharmacogenomic research, advance targeted therapies, and enhance cancer treatment development.

## Materials and Methods

### Overall pipeline design and implementation

**Figure 1:**
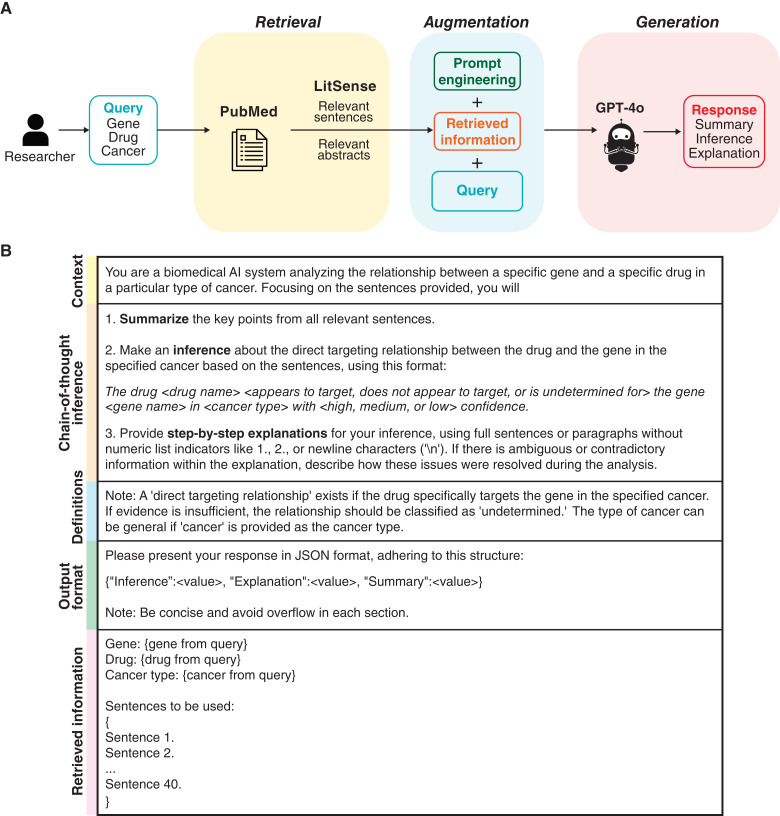
Automated LLM-based pipeline for drug–gene relationship inference. **A,** Pipeline design. The pipeline is designed to infer the relationship between a gene and a drug within a specific cancer type based on relevant literature. The process begins with a query containing a specific gene, drug, and cancer type. Relevant sentences or abstracts are extracted from PubMed using LitSense or the PubMed search engine, respectively. The retrieved information is then combined with a prompt as input for a LLM. The LLM generates a response including a summary of the retrieved information, an inference about the drug–gene relationship within the specified cancer context, and a detailed explanation. **B,** Prompt for inference based on sentence-level retrieval. The prompt structure includes context, chain-of-thought inference, definitions, output format, and retrieved information. The context section instructs the LLM to analyze drug–gene relationships in a specific cancer type. The chain-of-thought inference section outlines the steps for the LLMs to summarize retrieved information, infer the drug–gene relationship, and explain the inference process. The definition section clarifies key terms, such as drug–gene targeting relationships. The output format section specifies the response structure for automated result handling and downstream analysis. The prompt is merged with the query elements (gene, drug, and cancer type) and retrieved sentences. For abstract-level retrieval, “sentences” is replaced by “abstracts.” The alternative prompt designed to capture both direct binding and indirect modulation relationships is shown in Supplementary Fig. S1. AI, artificial intelligence.

We developed an automated pipeline designed to infer relationships between a drug and a gene, either within a specific cancer type or more broadly across various cancers. The pipeline is built around a LLM that utilizes a retrieval-augmented technique to improve inference accuracy by grounding its responses in current biomedical literature. Specifically, the LLM generates inferences based on relevant abstracts or sentences extracted from PubMed (RRID: SCR_004846) articles. As illustrated in Fig. 1A, the pipeline consists of three primary components: retrieval, augmentation, and generation. The process begins with the retrieval of relevant abstracts or sentences from PubMed that mention interactions between the specified gene and drug in a cancer context. In the augmentation step, the LLM is directed to incorporate this retrieved information when making inferences about the drug–gene relationship. Finally, the generation phase produces outputs that include an inference of the relationship, a rationale for the inference, and a summary of the supporting information retrieved from the literature. To enable full automation, the pipeline integrates the APIs of PubMed and various LLMs. Each component is described in detail in the following sections.

### Retrieval of literature with drug–gene–cancer information

For abstract retrieval, we used a keyword search approach by PubMed’s “Best Match” algorithm (5), which uses machine learning incorporating more than 150 relevance factors, such as article usage, term matching, article type, and publication date, to prioritize the most relevant articles (i.e., ranking articles by the “relevance” field). Specifically, we used queries in the format of “*<gene> AND <drug> AND <cancer type>*,” such as “*CDK4 AND palbociclib AND breast cancer*.” If a specific cancer type was not specified, we used “cancer” as a general query term to capture broader cancer-related literature. Our pipeline retrieved abstracts using the R rentrez library (PeerJ Preprints 2017.08.25.3179), which provides an interface to the Entrez Programming Utilities API developed by the NCBI to access PubMed (18). Because PubMed updates its database daily, our approach ensures access to the most up-to-date biomedical literature.

For sentence retrieval, we utilized LitSense (19), a specialized search tool indexing sentences from more than 36 million articles in PubMed and PubMed Central. LitSense ranks sentence relevance using a combination of inverse document frequency for term-based retrieval and text embeddings for semantic similarity to the search query. Using the LitSense API, we queried phrases in the format of “*Does <drug> target <gene> in <cancer type>?*”—for example, “*Does palbociclib target CDK4 in breast cancer?*” The top-ranked sentences were extracted for further analysis. Because LitSense regularly fetches updates from PubMed and PubMed Central, it ensures access to the latest sentence-level information.

### Proprietary and open-source LLMs

We evaluated six state-of-the-art LLMs in this study (Supplementary Table S1). GPT-4o (OpenAI) served as the primary model due to its superior performance in recent biomedical studies (medRxiv 2024.08.11.24311810; ref. 20). In addition, we evaluated another proprietary model, Gemini-1.5 Pro (Google; hereafter referred to as Gemini), and four open-source models, namely Llama-3-70B (Meta; Llama-3), Mixtral-8x7B (Mistral AI; Mixtral), Mistral-7B (Mistral AI; Mistral), and Llama-3.2-3B-PubMed (Llama-3.2-PubMed). Of note, Llama-3.2-PubMed is a Llama-3.2–based model fine-tuned specifically for biomedical applications using PubMed data. The proprietary models were accessed via their respective APIs. The open-source LLMs were downloaded and run using the Ollama platform on our local high-performance computing servers with 128 GB memory, 16 CPUs, and an L40S 48 GB GPU at the University of Pittsburgh Center for Research Computing. All models were run with their default parameter settings, including temperature control. The implementation of the baseline non-LLM BioBERT model (version BioBERT-Base-v1.1; ref. 21) is described in the Supplementary Methods.

### Prompt design, prompt engineering, and LLM input/output

The prompt serves as the text input that instructs the LLM to generate its output. We used prompt engineering techniques to ensure accurate and reliable responses. The prompt is organized into five sections: context, chain-of-thought inference, definition, output format, and retrieved information (Fig. 1B; Supplementary Fig. S1).

1. **Context:** The prompt begins by assigning the LLM the role of a biomedical artificial intelligence system, specifying its objective to analyze drug–gene relationships within a specific cancer type based on provided literature rather than relying on the internal knowledge of the LLM.
2. **Chain-of-thought inference:** The prompt uses a chain-of-thought strategy to guide the LLM through a logical and rational inference process (22). Specifically, we first instructed the LLM to summarize the key points from the retrieved information to ensure it comprehends the content. Next, it infers the drug–gene relationship with a confidence level. A core feature of the chain-of-thought strategy is to prompt the LLM to provide step-by-step explanations for the reasoning behind the inference. This technique has been shown to enhance reasoning capabilities (23). Additionally, we instructed the LLM to address how it resolves any ambiguous or contradictory information found in the retrieved data.
3. **Definitions:** We defined key terms, such as “direct targeting relationship” and “undetermined relationship,” in the context of drug–gene interactions in cancer.
4. **Output format:** We specified a structured response format to facilitate further analysis.
5. **Retrieved information:** The prompt ends by appending the retrieved abstracts or sentences to provide the LLM with relevant literature as evidence for the analysis.

This prompt is then fed into the LLM to generate responses. As instructed in the chain-of-thought inference section, the output includes three main components:

1 A summary of the retrieved information.
2 An inference regarding the relationship between the drug and the gene in cancer, accompanied by a confidence level. The inference follows the following format:
  “The drug <drug name> <appears to target, does not appear to target, or is undetermined for> the gene <gene name> in <cancer type> with <high, medium, or low> confidence.”
3 A step-by-step explanation of the inference process.

### Curated drug–gene relationships for performance evaluation

To evaluate the performance of the inference pipeline, we compiled a total of 539 positive and negative drug target cases from two databases: CancerDrugs DB and PharmGKB (RRID: SCR_002689). CancerDrugs DB is developed by the Anticancer Fund by collecting approved cancer drugs from various data sources (24). From this database, we extracted a total of 218 drugs that specifically target genes in particular cancer types, which served as positive drug–gene–cancer cases for the benchmark. Because some cases involved rare or highly specific cancer types, we recategorized the cancer types based on the primary sites, resulting in a total of 25 distinct cancer types. Negative drug–gene relationships were curated from PharmGKB (25, 26). We collected 321 drug–gene pairs annotated as “not associated” in PharmGKB, meaning these pairs were evaluated but not found to have statistically significant relationships. Due to the lack of specific disease annotations for these pairs, we used “cancer” as a general term in our pipeline to maintain focus on oncology. This approach aligns with the limited availability of documented negative cases while prioritizing likely true nonassociations (see “Discussion”).

### Performance evaluation metrics

For quantitative evaluation, LLM inferences were converted to scores from 1 to 9: 1 to 3 for nontarget, 4 to 6 for undetermined, and 7 to 9 for target relationships (Supplementary Fig. S2). Confidence levels (low, medium, and high) were integrated; for example, a score of 1 represents a high-confidence nontarget relationship, whereas 9 indicates a high-confidence target relationship. We utilized multiple performance metrics to assess the ability of the LLM to distinguish between positive and negative drug–gene cases. The primary metric was the area under the ROC curve (AUC), which reflects the model’s overall discrimination ability (27). The Youden index (28) was applied to determine the threshold score for each LLM in the comparative analysis. Additional evaluation metrics, including accuracy, sensitivity, specificity, precision, recall, F1 score, and κ values, were provided for a comprehensive assessment.

### Construction of a drug–gene interaction network

To construct a comprehensive drug–gene interaction network, we refined the prompt to infer two types of drug-targeting mechanisms: direct binding, in which a drug directly binds to the protein of a gene, and indirect modulation, in which a gene influences drug sensitivity or a drug affects gene expression (Supplementary Fig. S1). The pipeline was applied to pairwise combinations of 214 well-documented oncogenes from the Catalogue of Somatic Mutations in Cancer (RRID: SCR_002260; the tier-1 Cancer Gene Census collection; ref. 29) and 144 FDA-approved oncology drugs from the Repurposing Data Portal (30). The LLM was instructed to summarize the major cancer type(s) associated with each drug–gene interaction based on retrieved sentences. We categorized each interaction into 26 cancer groups: 25 major cancer types defined by primary sites, and an “other” category for less common cancer types. Each drug–gene pair could be assigned to multiple groups, reflecting its relevance across different cancer types. The identified drug–gene interactions were visualized and analyzed as a network using Cytoscape (RRID: SCR_003032; ref. 31).

### Development of the GeneRxGPT web tool

We developed GeneRxGPT, an interactive web tool designed to make the pipeline accessible. In addition to retrieving literature from PubMed and LitSense, GeneRxGPT utilizes the PubChemR and Webchem R libraries to obtain drug-related information from PubChem (RRID: SCR_004284), including drug structures, Simplified Molecular Input Line Entry System codes, and biochemical features. The web server was developed using R (v4.4.0) and R Shiny (v1.8.1; ref. 32). The httr (33) R library (v1.4.7) interfaced with the APIs of LitSense and proprietary LLMs. Additional primary R libraries for data processing and visualization included rcdk (34) and stringr. The web tool also provides access to the drug–gene interaction network with easy-to-use filtering and visualization tools. Detailed documentation and examples guide users through its features. The web server currently runs on VMWare infrastructure with 8 Intel cores and 64 GB of RAM, hosted at the University of Pittsburgh Center for Research Computing.

### Data availability

All datasets used in this study are accessible through the sources described in the previous sections, Supplementary Methods, and cited references. The developed pipeline and results are available via the GeneRxGPT web tool at https://shiny.crc.pitt.edu/generxgpt/. The source code is available on figshare (https://doi.org/10.6084/m9.figshare.27849816).

## Results

### Automated pipeline for literature-based inference of drug–gene relationships

We developed a retrieval-augmented approach to harness the capabilities of emerging LLMs for inferring drug–gene relationships in cancer. Rather than depending solely on the internal knowledge of LLMs, our approach integrates literature-based evidence to ensure that inferences are grounded in the most current, referenceable data. We used GPT-4o, the latest model released by OpenAI, as the primary model due to its superior performance in recent biomedical studies. The pipeline, as illustrated in Fig. 1A, interfaces programmatically with GPT-4o and PubMed and comprises three core components: (i) retrieval of relevant sentences or abstracts from PubMed that describe interactions between the drug, gene, and cancer type of interest; (ii) augmentation of LLM-generated inferences by incorporating this retrieved information; and (iii) generation of inference results.

The pipeline produces three main outputs: (i) a concise summary of the retrieved literature; (ii) an inference regarding the drug–gene relationship within a cancer context, with an associated confidence level; and (iii) a step-by-step explanation that clarifies how the retrieved literature supports the inference. By grounding the inference process in current literature, our pipeline enhances result transparency and interpretability, which are important for ensuring the robustness and reliability of drug–gene interaction exploration. We instructed the LLM to infer two types of drug–target relationships: (i) direct binding (for systematic performance evaluation; Fig. 1B) and (ii) indirect modulation (for constructing a drug–gene interaction network that includes both direct and indirect relationships; Supplementary Fig. S1).

### Identification of well-studied and misclassified drug targets

**Figure 2:**
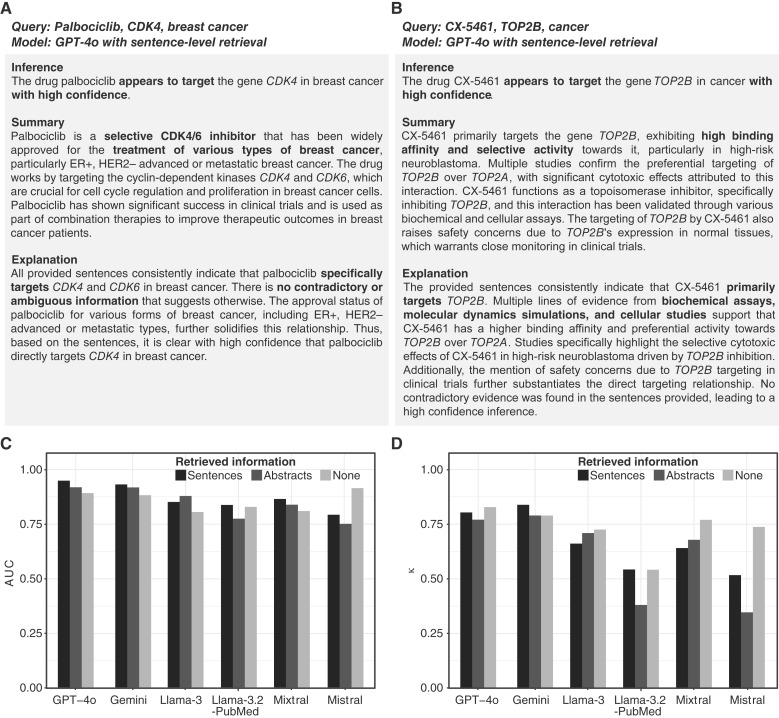
Example pipeline outputs and performance evaluation across retrieval approaches and LLMs. **A,** Pipeline output for an established drug–gene relationship between palbociclib and *CDK4* in breast cancer. **B,** Output for CX-5461, a drug with a historically misclassified target, using sentence-level retrieval. CX-5461 was previously misclassified as targeting RNA polymerase I until evidence from 2021 confirmed *TOP2B* as the primary target. Sentence-level retrieval enabled the pipeline to correctly identify *TOP2B* with high confidence, whereas GPT-4o without retrieval did not recognize this target (see Supplementary Fig. S4), likely due to outdated information. **C** and **D,** Comparison of AUC and κ statistics across LLMs and retrieval methods. Bar plots show the performance of six different LLMs (GPT-4o, Gemini, Llama-3, Llama-3.2-PubMed, Mixtral, and Mistral) using sentence retrieval, abstract retrieval, and without retrieval (relying solely on LLM knowledge). The κ statistics measures the agreement between LLM inference and the two databases. Detailed results are available in Supplementary Tables S4–S6.

To illustrate the effectiveness of our pipeline in identifying pharmacogenomic relationships, we began by testing several well-known drug targets. For instance, palbociclib is a chemotherapeutic agent and the first *CDK4* inhibitor approved for breast cancer (35). Our pipeline summarized sentences retrieved from PubMed concerning palbociclib, *CDK4*, and breast cancer and correctly stated that palbociclib has been approved for metastatic estrogen receptor/progesterone receptor–positive, HER2-negative breast cancer (Fig. 2A; ref. 36). It correctly inferred that palbociclib targets *CDK4* in breast cancer with high confidence. We then assessed the capability to identify nonassociations, such as metformin and *BRCA2*. The pipeline retrieved sentences discussing the targeting of *BRCA2* in breast cancer by various drugs, including PARP inhibitors and *mTORC1* (37), but found no evidence linking metformin to *BRCA2*. Consequently, the inference reported with high confidence that metformin does not target *BRCA2* in breast cancer (Supplementary Fig. S3).

We further examined challenging cases involving drugs whose primary mechanisms have been refined by new evidence. One such case was CX-5461, which was initially characterized as targeting RNA polymerase I transcription (38). However, emerging evidence published in 2021 identified *TOP2B* as its primary target, raising potential safety concerns (39). When querying GPT-4o directly during our study in October 2024 (version 2024-05-13) without any retrieved literature, the model incorrectly inferred that CX-5461 does not target *TOP2B* in cancer (Supplementary Fig. S4), likely due to outdated information within its internal knowledge base. In contrast, our pipeline, even when presented with mixed information from both older and recent studies, correctly identified that CX-5461 targets *TOP2B* in cancer with high confidence (Fig. 2B). A similar case was observed with OTS964, a drug initially developed to target the protein kinase *TOPK* (40) but later shown to primarily target *CDK11* (41, 42). Our pipeline correctly inferred that OTS964 targets *CDK11* with high confidence, whereas GPT-4o alone incorrectly identified *TOPK* as the main target (Supplementary Fig. S5). These examples, covering well-known, nonassociated, and misclassified drug targets, demonstrate the robustness of our pipeline based on current literature.

### Systematic performance evaluation for optimizing information retrieval

We systematically evaluated the ability our pipeline to distinguish targeting (positive) and nonassociating (negative) drug–gene relationships. For this assessment, we compiled a dataset of 539 well-documented drug–gene cases from CancerDrugs DB and PharmGKB, including 218 positive and 321 negative cases as ground truth. To focus the evaluation, we specifically instructed GPT-4o to infer direct targeting relationships (Fig. 1B). The inference results were converted into scores to quantitatively assess performance (see “Materials and Methods”; Supplementary Fig. S2).

A critical aspect of this evaluation was to determine the optimal amount of retrieved information to maximize performance, as retrieving more data could either provide useful context or introduce irrelevant information. After testing retrievals ranging from 1 to 100 sentences, we found that retrieving the top 40 sentences yielded the highest AUC of 0.94 (Supplementary Fig. S6A; Supplementary Table S2). On average, our pipeline analyzed each drug–gene–cancer case in 4.3 seconds at a cost of $0.014, based on API rates as of October 2024. This represents a 56-fold increase in speed over human reading time (43). Similarly, we analyzed the numbers of retrieved abstracts and found that the top 10 most relevant abstracts produced the best results (AUC = 0.91; Supplementary Fig. S6B; Supplementary Table S3).

### Performance comparison across various LLMs

We further compared GPT-4o with five other state-of-the-art LLMs across sentence-level, abstract-level, and no retrieval. GPT-4o with sentence retrieval achieved the highest performance overall (AUC = 0.94; Fig. 2C), surpassing its own abstract-level (AUC = 0.91) and non-retrieval (AUC = 0.88) methods. The other proprietary model, Gemini, performed comparably well, with sentence, abstract, and no retrieval yielding AUCs of 0.92, 0.90, and 0.87, respectively. Interestingly, Mistral, an open-source LLM, performed better without retrieval, achieving an AUC of 0.91 (Fig. 2C).

We also used κ values to assess the agreement between inferred drug–gene relationships and the ground truth. Gemini and GPT-4o demonstrated the highest agreement (κ values, 0.84 and 0.80; Fig. 2D) when using sentence-level retrieval (Supplementary Tables S4–S6). In contrast, Llama-3.2-PubMed and Mistral performed the worst with abstract-level retrieval (κ values, 0.38 and 0.35). Overall, our analysis highlighted the effectiveness of sentence-level retrieval across most models. Additional performance metrics are provided in Supplementary Tables S4–S6.

As a baseline, we fine-tuned BioBERT (21), a non-LLM method, for extracting drug–gene relations from retrieved sentences (See Supplementary Methods). BioBERT was evaluated on a simplified binary classification (target vs. not) without considering cancer context and showed limited performance (AUC = 0.55; Supplementary Table S4). Aggregating sentence-level results at the drug–gene level further yielded a low κ value of 0.11. These findings highlight the need for advanced LLMs to analyze complex drug–gene relationships.

### Evaluation of confidence levels and consistency in GPT-4o inference

Based on performance evaluation results, our final pipeline was implemented using GPT-4o with sentence-level retrieval. In addition to target/nontarget inference, GPT-4o also assigned a confidence level (high, medium, or low) to each prediction. To assess the reliability of the confidence levels, we conducted a contamination test on 50 positive and 50 negative drug–gene–cancer cases. For each case, we progressively replaced the 40 relevant retrieved sentences with irrelevant ones randomly sampled from arXiv and analyzed how the outputs changed (see Supplementary Methods). When all 40 sentences were irrelevant, inferences were undetermined with low confidence (Supplementary Fig. S7). Adding a single relevant sentence typically shifted the inference to target, with confidence levels increasing as more relevant sentences were included (Supplementary Fig. S7A). A similar trend was observed for negative cases (Supplementary Fig. S7B), confirming that the confidence levels meaningfully reflect the relevance of retrieved evidence.

We also evaluated the consistency of GPT-4o responses by repeating the same input query five times (Supplementary Methods). Overall, the high intraclass correlation coefficient (0.86; 95% confidence interval: 0.84–0.88) indicates strong reliability despite inherent generative variability in LLMs.

### Mapping a pan-cancer drug–target interaction network

We utilized our pipeline to develop a comprehensive map of drug–gene interactions to uncover potential therapeutic targets and explore drug repurposing opportunities. This process involved analyzing pairwise relationships between 144 FDA-approved oncology drugs and 214 well-documented oncogenes from COSMIC. For this analysis, we refined our prompt to assess both direct binding and indirect modulation relationships for each drug–gene pair (Supplementary Fig. S1). The pipeline also annotated each pair with the associated major cancer type(s) based on retrieved sentences.

**Figure 3:**
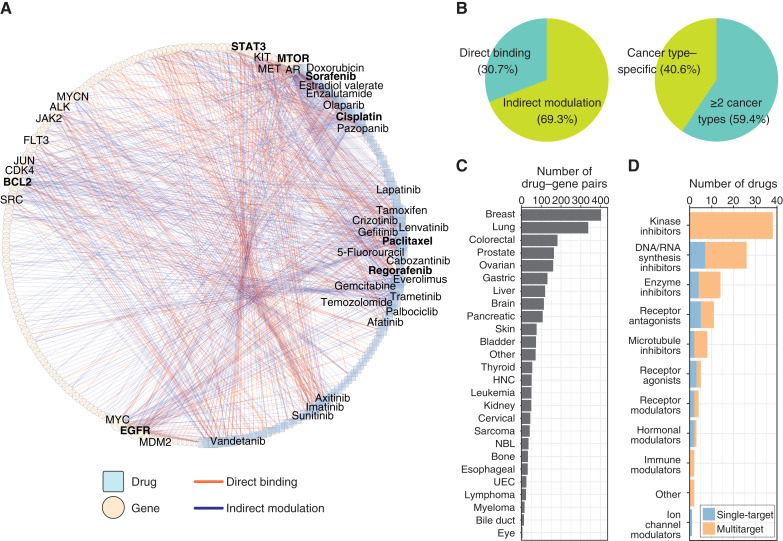
Pan-cancer drug–target interaction network. **A,** The network includes 854 drug–gene pairs identified by our pipeline through pairwise combinations of 214 oncogenes and 144 FDA-approved oncology drugs as high-confidence drug–gene interactions. For each identified pair, the pipeline summarizes the major cancer type(s) based on which the inference was made from the retrieved information. In the network, circles and squares represent drugs and genes, respectively. Edge colors indicate the type of interaction. Node sizes are proportional to the degree of connectivity (i.e., the number of interactions), with names labeled for genes and drugs with more than 10 connections. Drugs and genes discussed in the main text are highlighted in bold. **B,** Distributions of interaction types (direct binding or indirect modulation) and cancer type specificity (interactions present in a single or multiple cancer types). **C,** Distribution of cancer types across 25 primary sites and an additional “other” group of all other cancers. Note that each drug–gene interaction may be associated with multiple cancer types. HNC, head and neck cancer; NBL, neuroblastoma; UEC, uterine/endometrial cancer. **D,** Distribution of drug mechanisms of action and target specificity, which categorizes drugs as single-target (interacting with only one of the 214 genes) or multitarget (interacting with multiple genes).

Our pipeline identified 854 high-confidence drug–gene interaction pairs, involving 114 drugs and 154 genes (Fig. 3A; Supplementary Table S7). Of these pairs, 30.7% (*n* = 262) were direct binding relationships, whereas 69.3% (*n* = 592) involved indirect modulation (Fig. 3B), likely reflecting the complexity of indirect interactions. In the constructed pan-cancer drug–gene network, the top hub gene of the network was *mTOR*, which interacted with 45 drugs, followed by *BCL2*, *STAT3*, and *EGFR* (Fig. 3A; Supplementary Table S8). These genes are central to key cellular processes like proliferation and survival, underscoring their importance in cancer therapies.

Next, we assessed the cancer type specificity of these interactions. On average, each drug–gene pair was associated with 2.8 cancer types. As shown in Fig. 3B, only 347 pairs (40.6%) were cancer type–specific. This distribution suggests that most drug–gene interactions span multiple cancer types and highlights shared molecular pathways. Breast and lung cancers had the most drug–gene pairs (403 and 338, respectively), reflecting prevalent cancers with extensive therapeutic development and research interest. In contrast, eye cancer, bile duct cancer, and myeloma had the fewest (4, 12, and 15, respectively; Fig. 3C).

Our analysis also revealed drugs with high connectivity in the network (Supplementary Table S9). Cisplatin, a DNA/RNA synthesis inhibitor, emerged as the top hub drug (interacting with 44 genes), followed by kinase inhibitors sorafenib and regorafenib and a microtubule inhibitor paclitaxel. These results underscore the broad versatility of these drugs in interacting with multiple genes. Most drugs (77.2%; *n* = 88) exhibited multitarget profiles. In particular, all 38 kinase inhibitors interacted with multiple genes (Fig. 3D), reflecting their polypharmacologic potential (44). Similarly, immune modulators (100.0%; *n* = 2) and chemotherapy agents, including DNA/RNA synthesis inhibitors (73.1%; *n* = 26) and microtubule inhibitors (75.0%; *n* = 8), showed extensive multitarget interactions. This finding aligns with the broad mechanisms of action of these drugs within complex cellular and immunologic contexts. In contrast, targeted therapeutic approaches, such as receptor agonists, hormonal modulators, and the ion channel modulator, demonstrated a higher proportion of single-target specificity (60.0%, 66.7%, and 100.0% for 5, 3, and 1 drugs, respectively). In summary, our pan-cancer network provides a detailed landscape of direct and indirect drug–target relationships, highlighting the versatility of certain drugs across cancers and genes.

### Identification and validation of *CTNNB1* mutations in sorafenib sensitivity modulation in liver cancer

**Figure 4:**
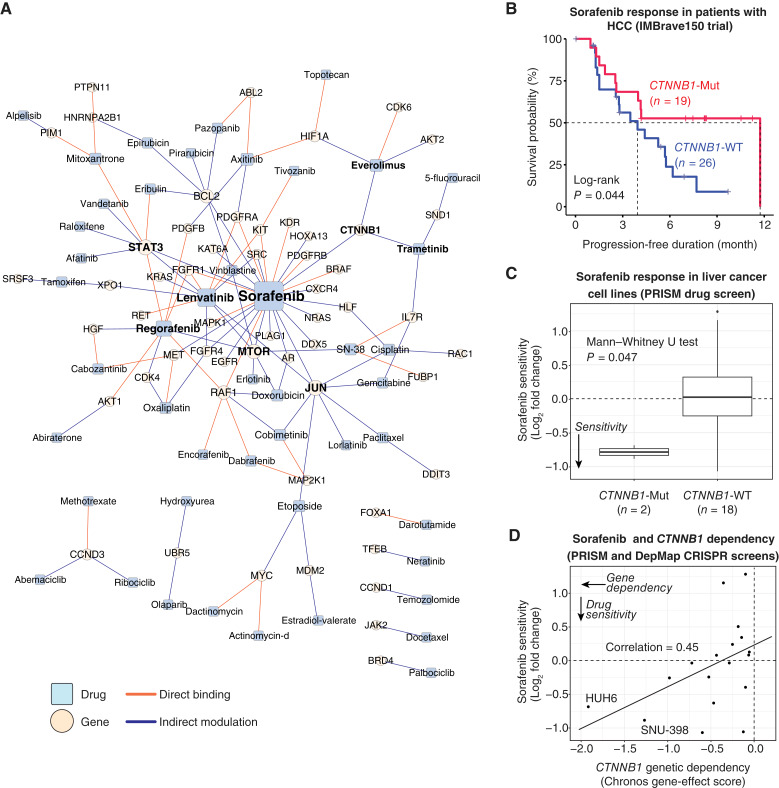
Drug–target network in liver cancer. **A,** Drug–target network in liver cancer. The network was constructed by extracting drug–gene pairs associated with liver cancer from Fig. 3A, in which liver cancer was identified as a primary cancer type among multiple possible types generated through GPT-4o inference. The node size and font size are proportional to the degree (i.e., the number of connections). Drugs and genes discussed in the main text are highlighted in bold. **B,** Kaplan–Meier curves for progression-free survival of patients with HCC, the major type of liver cancer, treated with sorafenib in the IMBrave150 clinical trial, comparing *CTNNB1*-mutated (*CTNNB1*-Mut; *n* = 19) and *CTNNB1* wild-type patients (*CTNNB1*-WT; *n* = 26). Statistical significance was assessed by the log-rank test. **C,** Boxplots showing differential sensitivity to sorafenib between *CTNNB1*-mutated (*CTNNB1*-Mut; *n* = 2) and *CTNNB1* wild-type liver cancer cell lines (*CTNNB1*-WT; *n* = 18). Drug sensitivity data are represented as the log_2_ fold change in cell counts following treatment, derived from the Profiling Relative Inhibition Simultaneously in Mixtures (PRISM) drug screening project. Statistical significance was assessed by the one-tailed Mann–Whitney U test. **D,** Correlation between *CTNNB1* genetic dependency (represented by the Chronos gene-effect score from DepMap) and sorafenib sensitivity (PRISM) in liver cancer cell lines (*n* = 18). A more negative gene-effect score represents a stronger genetic dependency. The names of cell lines exhibiting strong dependency on *CTNNB1* (with gene-effect scores < −1) are labeled.

Liver cancer is the third leading cause of cancer-related deaths worldwide, underscoring the urgent need for novel therapeutic strategies (16, 17). To investigate potential solutions, we extracted 119 drug–gene pairs (47 drugs and 55 genes) associated with liver cancer from the pan-cancer network (Fig. 4A). Sorafenib exhibited the highest number of gene interactions. This multikinase inhibitor is the first FDA-approved drug for hepatocellular carcinoma (HCC), the most common type of liver cancer in adults. It interacted with 25 genes (17 through indirect modulation and 8 through direct binding). Following sorafenib, lenvatinib, another approved drug for HCC, emerged as an important multitarget kinase inhibitor, with seven indirect and four direct gene interactors. Within the network, *STAT3*, *JUN*, and *MTOR* were identified as the top hub genes, with relationships to 8, 8, and 7 drugs, respectively. These genes have pivotal roles in liver cancer therapeutics due to their roles in regulating key pathways involved in cell proliferation, survival, and metabolism (45–47).

We turned our attention to *CTNNB1*, the sixth-ranked hub gene in our constructed network and one of the most frequently mutated genes in liver cancer (48). Directly targeting *CTNNB1* with drugs has proven challenging (49), and patients with mutated *CTNNB1* often respond poorly to the standard-of-care immune checkpoint inhibitors (50). Aligning with current knowledge, *CTNNB1* showed no direct targeting relationship with any drug in the network. However, it demonstrated high-confidence indirect interactions with sorafenib, trametinib, and everolimus, indicating a potential therapeutic benefit of these drugs for this subset of patients with HCC. To validate this observation, we focused on sorafenib due to its established clinical use and the availability of extensive data from large-scale studies. Specifically, we reanalyzed data from the IMBrave150 phase 3 clinical trial in HCC (see Supplementary Methods; ref. 51). Kaplan–Meier curves showed significantly improved progression-free survival in *CTNNB1*-mutated patients treated with sorafenib compared with those with wild-type *CTNNB1* (Fig. 4B; *n* = 19 vs. 26; log-rank test *P* = 0.044), suggesting a meaningful modulation of treatment response. However, no significant difference was observed in overall survival, likely due to alternative pathways or resistance mechanisms.

To further isolate the effects of sorafenib and *CTNNB1* in a controlled setting with clearer genetic backgrounds, we examined drug sensitivity data (52) and CRISPR knockout screening results among liver cancer cell lines from Cancer Dependency Map (DepMap; Supplementary Methods; ref. 53). Indeed, *CTNNB1*-mutated cell lines showed significantly greater sensitivity to sorafenib compared with wild-type cells (Fig. 4C; *n* = 2 vs. 18; one-tailed Mann–Whitney U test *P* = 0.047). These findings support the role of *CTNNB1* mutations in modulating sorafenib response. Additionally, we observed a positive correlation (Pearson correlation coefficient = 0.45; *n* = 18) between the reduction in cancer cell viability induced by sorafenib treatment and that caused by *CTNNB1* knockout. This suggests that cells more dependent on *CTNNB1* are also more responsive to sorafenib treatment (Fig. 4D). In summary, our findings demonstrate that *CTNNB1* mutations are associated with enhanced sensitivity to sorafenib in liver cancer, highlighting a potential therapeutic strategy for targeting this vulnerable patient subset.

### GeneRxGPT: a user-friendly web tool for the inference pipeline

**Figure 5:**
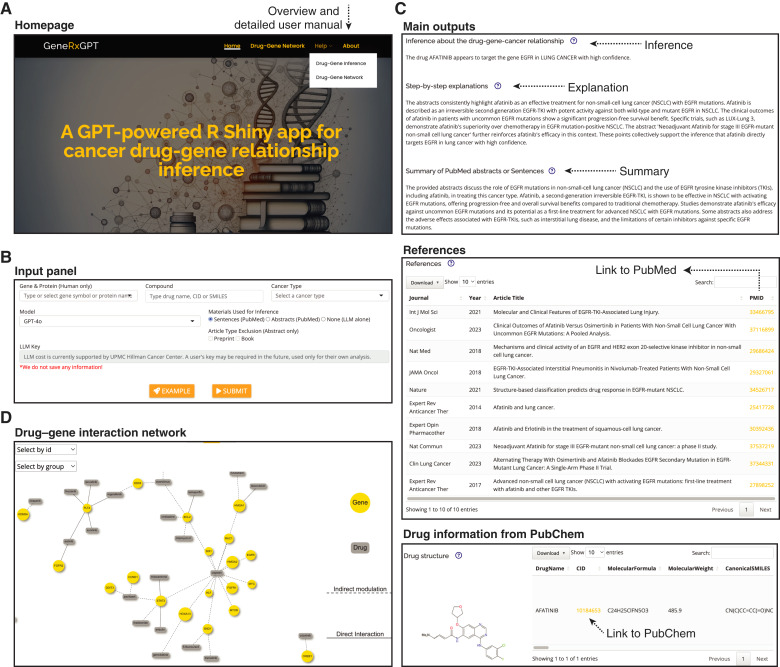
The GeneRxGPT web tool. **A,** Homepage. The homepage provides access to the main drug–gene inference module, as well as a link to the drug–gene network module. Users can easily test the tool using a built-in example. A comprehensive user manual, along with figure and table legends, is available on the help pages or by clicking the question mark icon in individual output sections. **B,** Input panel of the drug–gene inference module. Users enter a gene (gene symbol or protein name), drug [name, PubChem CID, or Simplified Molecular Input Line Entry System (SMILES) code], and cancer type into the input panel. Users can select the desired LLMs (either GPT-4o or Gemini-1.5 Pro, currently available) and choose whether the inference is based on sentence- or abstract-level retrieval. **C,** Output panels of the drug–gene inference module. Based on the abstracts or sentences retrieved from relevant publications in PubMed, the selected LLM generates an inference and detailed explanation of the drug–gene relationship in the specified cancer type, along with a summary of the retrieved information. References to retrieved sentences or abstracts are provided with PubMed PMIDs and hyperlinks. Additionally, detailed drug information from PubChem is presented by an informative table and the drug structure. **D,** Drug–gene interaction network. This module allows users to visualize a drug–target interaction network among 214 oncogenes and 144 FDA-approved drugs, either across all cancers or within a specific cancer type.

To enhance accessibility for cancer researchers, we developed GeneRxGPT, an intuitive R Shiny web application that provides easy access to the pipeline and drug–gene network without requiring programming skills or extensive computational resources (Fig. 5A). The tool interfaces with PubMed, PubChem, and LLMs through built-in APIs. It is hosted on our institutional high-performance computing infrastructure and can be accessed at https://shiny.crc.pitt.edu/generxgpt/. A comprehensive overview, detailed user manual, and input examples are provided on the help page (Fig. 5A).

GeneRxGPT features two main modules: **(i) drug**–**gene inference** and **(ii) drug**–**gene network**, which run the literature-based pipeline and visualize the pan-cancer drug–gene network, respectively. The **drug**–**gene inference** module leverages the automated pipeline to infer drug–gene targeting relationships based on real-time literature retrieval. Researchers can simply input a gene (gene symbol or protein name) and a drug (name, Simplified Molecular Input Line Entry System code, or PubChem CID) and select a cancer type through the input panel (Fig. 5B). Users can choose from the two best-performing LLMs, GPT-4o and Gemini, and specify their preferred retrieval method (sentences or abstracts) and exclusion criteria (e.g., preprints). GeneRxGPT first retrieves detailed information about the query drug and provides a direct URL link to its profile in PubChem (Fig. 5C). The tool then compiles the retrieved sentences or abstracts into a prompt and submits it to the selected LLM for analysis. As shown in Fig. 5C, the output includes a concise one-sentence inference regarding the drug–gene relationship, a detailed explanation of the inference process, and a summary of the retrieved information. Importantly, GeneRxGPT ensures transparency by providing references to all sources used in the inference, including journal names, publication years, titles, and PubMed PMIDs with direct URLs. The **drug**–**gene network** module allows users to interactively examine the pan-cancer drug–gene interaction network (Fig. 5D). Users can view the pan-cancer network, generate a subnetwork associated with a specific cancer type, and filter the network by confidence level (high, medium, or low). Additionally, the tool provides an operable and downloadable table with detailed information on the genes and drugs.

In summary, GeneRxGPT provides a streamlined, accessible platform that integrates state-of-the-art LLMs with real-time literature retrieval. We anticipate that this tool will empower cancer researchers to efficiently explore and interpret drug–gene relationships.

## Discussion

The vast and ever-growing volume of biomedical literature has led to information overload in drug–gene studies, making it increasingly difficult for researchers to stay updated and synthesize relevant insights efficiently (54, 55). Emerging LLMs offer promising solutions, given their unprecedented ability to digest and interpret complex pharmacogenomic information (56, 57). In this article we introduce, to the best of our knowledge, the first retrieval-augmented pipeline using GPT-4o to infer relationships between genes and drugs in cancer research. By integrating real-time information from PubMed, our approach addresses a significant limitation of traditional static LLMs, which often rely on outdated knowledge and can generate inaccurate or misleading inference on drug–gene relationships. For example, our analysis successfully corrected the mischaracterizations that occurred when using the static GPT-4o model alone for CX-5461 and OTS964. This discrepancy, partly due to outdated internal data within GPT-4o, highlights the critical importance of real-time literature retrieval in pharmacogenomics research. Additionally, the issue of false PMIDs generated by LLMs (14) underscores the advantage of using our retrieval-augmented approach, which grounds outputs in verifiable and current sources.

Our pipeline enabled an efficient exploration among hundreds of approved drugs and key oncogenes. Network analysis identified hub genes (e.g., *mTOR*, *BCL2*, and *STAT3*) and hub drugs (e.g., cisplatin, sorafenib, and regorafenib), revealing key therapeutic strategies. Our case study in liver cancer, particularly on the interaction between *CTNNB1* mutations and sorafenib, exemplifies the practical utility of the pipeline. Follow-up literature review confirmed this correlation in patients with HCC (51, 58). Beyond its known targets such as *VEGFR* and *RAF* (59), sorafenib also affects the WNT signaling pathway in *CTNNB1*-mutant HCC (60, 61). Studies have shown that sorafenib reduces β-catenin levels and downregulates WNT signaling in *CTNNB1*-mutant HCC cells and animal models, likely through interactions with other cellular pathways including TGFβ (60). Our findings were further validated by data from a clinical trial and drug sensitivity assays. These findings support the hypothesis that *CTNNB1*-mutant tumors may exhibit enhanced sensitivity to sorafenib, offering potential therapeutic benefit for this subset of patients.

Despite its strengths, our pipeline has limitations that warrant further development. The current prompts are tailored to identify direct and indirect interactions. However, the adaptability of the pipeline allows for the expansion of prompts to accommodate more complex interaction and gene mutation types, should future research require a deeper or more specific analysis of drug–gene dynamics. Beyond retrieval-based methods, creating domain-specific models based on fine-tuning may be a promising alternative. However, our results show that both fine-tuned LLM (Llama-3.2-PubMed) and non-LLM (BioBERT) underperformed compared with other models. Further studies are warranted to evaluate fine-tuning strategies for targeted biomedical tasks using larger annotated datasets and improved optimization. Another key issue is the inclusion of irrelevant information in the retrieved data, which can introduce noise and affect inference accuracy. Although our pipeline uses embedding-based search engines (LitSense) and keyword searches (PubMed search engine) to filter relevant data efficiently, some irrelevant content may persist. To mitigate this, our method assigns a confidence level to each inference. We verified these confidence levels using a contamination approach. However, more robust content filtering mechanisms may be needed to address irrelevant or even fabricated information (62). To this end, emerging multiagent systems that detect and assess the relevance of retrieved data (arXiv 2024.10.08.2405.18111) could be integrated into future versions of our pipeline.

Hallucination is a critical limitation of LLMs (12). Our pipeline leverages retrieval-augmented generation to enhance factual accuracy by grounding outputs in real-time information from authoritative sources (arXiv 2024.01.08.2401.01313; arXiv 2021.04.15.2104.07567). We used a multipronged approach: real-time data and knowledge retrieval from the literature, prompt engineering to ensure well-grounded responses, and rigorous evaluation of generated inferences against established drug–gene relationships. These measures ensure that inferred drug–gene relationships are not only up-to-date but also scientifically sound, as demonstrated by the systematic evaluation of inferences. This benchmarking process validates the accuracy of the pipeline’s outputs and directly assesses the risk of hallucination. However, whereas the inferences are grounded in robust evaluation, the explanations and free-text summaries derived from the retrieved data remain more vulnerable to hallucination, particularly in cases in which incomplete or ambiguous data affect rare or poorly studied interactions. To address this challenge, future improvements could include multiagent systems to collaboratively filter retrieved content and verify generated results (arXiv 2024.05.25.2405.16205) and hallucination detection methods (63) to further enhance the reliability of explanatory text and summaries (12). These enhancements will be essential for maintaining accuracy and trustworthiness as the complexity of biomedical research continues to grow.

The selection and curation of negative drug–gene relationships for evaluation posed a challenge due to the scarcity of well-documented negative interactions in databases. PharmGKB served as our primary source, with “not associated” pairs representing relationships evaluated in the literature but lacking statistically significant associations. These cases were selected for their likely reliability as true negatives. However, the absence of specific cancer annotations required us to generalize the disease focus to “cancer,” which may underrepresent certain interactions and introduce bias favoring well-studied drugs and genes. Future expansions could incorporate broader resources, such as like DGIdb (3), that may offer a more comprehensive range of interactions.

Our pipeline relies on PubMed as the primary source for information retrieval, but its coverage is not exhaustive. Expanding to include clinical trial repositories, pharmacokinetic/pharmacodynamic databases, high-throughput genetic and drug screens, and multi-omics datasets could enhance drug–gene interaction analysis (64). Additionally, structured data formats like knowledge graphs may enrich contextual information and improve model performance. Another challenge is managing context window limits and computation costs, as our prompts include up to 40 sentences or 10 abstracts, potentially leading to truncation in some LLMs. Future improvements could focus on summarization and selective synthesis to optimize efficiency and cost-effectiveness.

## Supplementary Material

Supplementary Methods Supplementary Methods

Table S1 Supplementary Table S1

Table S2 Supplementary Table S2

Table S3 Supplementary Table S3

Table S4 Supplementary Table S4

Table S5 Supplementary Table S5

Table S6 Supplementary Table S6

Table S7 Supplementary Table S7

Table S8 Supplementary Table S8

Table S9 Supplementary Table S9

Figure S1 Supplementary Figure S1

Figure S2 Supplementary Figure S2

Figure S3 Supplementary Figure S3

Figure S4 Supplementary Figure S4

Figure S5 Supplementary Figure S5

Figure S6 Supplementary Figure S6

Figure S7 Supplementary Figure S7
