# Supplementary material for: Inferring Drug–Gene Relationships in Cancer Using Literature-Augmented Large Language Models
Source: Cancer Res Commun. 2025 Apr 28;5(4):706–18. doi: 10.1158/2767-9764.CRC-25-0030 (PMC12036822; doi:10.1158/2767-9764.CRC-25-0030)
Supplement: Supplementary Methods [file crc-25-0030_supplementary_methods_suppsm.docx]

**Supplementary Methods**

**Validation datasets for liver cancer analysis**

We downloaded high-throughput CRISPR and drug screening data for liver cancer cell lines from the Cancer Dependency Map (DepMap) using its Data Explorer web tool. CRISPR loss-of-function screens were originally performed by DepMap (version 24Q2) (1). Drug screening data were generated by the Profiling Relative Inhibition Simultaneously in Mixtures (PRISM; version Repurposing Public 24Q2) project (2). For patient data, we collected sorafenib response data, *CTNNB1* mutation status, and survival data from the IMBrave150 phase 3 clinical trial in hepatocellular carcinoma (HCC) (3). A total of 130 patients underwent FoundationOne sequencing for alterations in 324 genes, including *CTNNB1*. Of these, we analyzed 45 patients treated with sorafenib for the survival analysis.

**Assessment of consistency in GPT-4o responses**

As a generative model, GPT-4o’s outputs can vary due to inherent randomness, making it essential to assess the consistency of its results. We evaluated this using the sentence-level retrieval pipeline. For each drug-gene-cancer case, we retrieved the top 40 relevant sentences from PubMed and kept them fixed throughout the analysis. Using these fixed sentences, we ran the pipeline five times per pair to generate multiple responses from GPT-4o under identical input conditions. We converted each output to a numeric score ranging from 1 to 9 (see Materials and Methods in the main text and Supplementary Fig. S2). To quantify consistency, we computed the intra-class correlation coefficient (ICC) based on these scores. A high ICC indicates strong consistency and robustness of our pipeline across repeated runs, despite the inherent variability of the LLM.

**Evaluation of confidence levels in GPT-4o inference using a** **contamination test**

Our pipeline uses GPT-4o to identify the relationship between a gene and a drug in the context of cancer. With each inference, we instructed GPT-4o to also assign a confidence level (high, medium, low). To assess the reliability of these confidence levels, we applied a contamination test in the sentence-level retrieval setting. For each drug-gene pair, we randomly selected 40 irrelevant sentences from arXiv preprints containing the keyword “cancer” but lacking information about the specific drug-gene-cancer pair being tested. We incrementally replaced the 40 relevant sentences retrieved from PubMed with these irrelevant ones, varying the number of replacements from 0 to 40. This allowed us to evaluate how GPT-4o’s outputs changed as the content progressively shifted from entirely irrelevant to entirely relevant. We conducted this analysis on randomly selected positive and negative drug-gene cases (*n* = 50 per group) and measured changes in the inference along with confidence levels (represented as scores from 1-9; see Supplementary Fig. S2) based on the proportion of relevant sentences.

**Non-LLM relation extraction model**

We included a traditional, non-LLM relation extraction model as a baseline for comparison with LLMs. Relation extraction refers to identifying semantic relationships between entities within a sentence. For this purpose, we employed BioBERT-Base-v1.1, a domain-specific adaptation of Bidirectional Encoder Representations from Transformer (BERT) pre-trained on over one million PubMed articles (4) (Supplementary Table S1). We fine-tuned the model on our retrieved sentences to tailor it for drug-gene relationship analysis. Due to technical limitations, we performed a simplified binary classification task to determine whether a sentence corresponded to a positive or negative case, regardless of cancer type. The BioBERT base model and fine-tuning code were obtained from <https://github.com/dmis-lab/biobert>.

Our sentence-level retrieval yielded 21,560 sentences covering 218 positive and 321 negative drug-gene pairs, with 40 retrieved sentences per pair. Sentences from positive cases were labeled as target, while those from negative cases were labeled as non-target. We applied five-fold cross-validation by randomly splitting all the sentences into five subsets, using four subsets for fine-tuning and one for testing. Relation extraction was conducted at the sentence level, assigning probabilities to indicate positive or negative relationships. The output probabilities in the testing set were evaluated using the AUC. The optimal threshold was determined using Youden’s index, and additional performance metrics were calculated accordingly. For evaluation at the drug-gene pair level, we aggregated sentence-level predictions of each pair based on majority voting.

**Supplementary References**

1. Tsherniak A, Vazquez F, Montgomery PG, Weir BA, Kryukov G, Cowley GS, et al. Defining a Cancer Dependency Map. Cell. 2017;170(3):564-76 e16.

2. Corsello SM, Nagari RT, Spangler RD, Rossen J, Kocak M, Bryan JG, et al. Discovering the anti-cancer potential of non-oncology drugs by systematic viability profiling. Nat Cancer. 2020;1(2):235-48.

3. Zhu AX, Abbas AR, de Galarreta MR, Guan Y, Lu S, Koeppen H, et al. Molecular correlates of clinical response and resistance to atezolizumab in combination with bevacizumab in advanced hepatocellular carcinoma. Nat Med. 2022;28(8):1599-611.

4. Lee J, Yoon W, Kim S, Kim D, Kim S, So CH, et al. BioBERT: a pre-trained biomedical language representation model for biomedical text mining. Bioinformatics. 2020;36(4):1234-40.
