# Supplementary material for: Inferring Drug–Gene Relationships in Cancer Using Literature-Augmented Large Language Models
Source: Cancer Res Commun. 2025 Apr 28;5(4):706–18. doi: 10.1158/2767-9764.CRC-25-0030 (PMC12036822; doi:10.1158/2767-9764.CRC-25-0030)
Supplement: Table S1 — Supplementary Table S1 [file crc-25-0030_table_s1_suppst1.pdf]

Supplementary Table S1. Overview of models (sorted by release date)

Model	Type	Version	Developer/ source	Access platform	Release date
Llama-3.2-3B-PubMed	LLM	Base: Llama-3.2-3B<br>Fine-tune: pubMedQA-finalDecision	--	Ollama (kronos483/Llama-3.2-3B-PubMed)	December 2024
Gemini	LLM	Gemini-1.5-pro-latest	Google AI	Google AI	May, 2024
GPT-4o	LLM	gpt-4o-2024-05-13	OpenAI	OpenAI	May, 2024
Llama-3	LLM	Meta-Llama-3-70B-Instruct	Meta	Ollama (llama3:70b)	April, 2024
Mixtral	LLM	Base: Mixtral 8x7B v0.1<br>Fine-tune: Instruct	Mistral AI	Ollama (mixtral:instruct)	January, 2024
Mistral	LLM	Mistral-7B-Instruct-v0.3	Mistral AI	Ollama (mistral:v0.3)	October, 2023
BioBERT-Base-v1.1	Non-LLM	Base: BioBERT-v1.1<br>Fine-tune (original): PubMed<br>Fine-tune (this study): Sentence retrieval for curated drug-gene pairs	DMIS Laboratory	GitHub (dmis-lab/biobert)	October, 2019
