# Supplementary material for: Inferring Drug–Gene Relationships in Cancer Using Literature-Augmented Large Language Models
Source: Cancer Res Commun. 2025 Apr 28;5(4):706–18. doi: 10.1158/2767-9764.CRC-25-0030 (PMC12036822; doi:10.1158/2767-9764.CRC-25-0030)
Supplement: Table S2 — Supplementary Table S2 [file crc-25-0030_table_s2_suppst2.pdf]

Supplementary Table S2. Evaluation of various numbers of retrieved sentences for model performance

Number of sentences	Accuracy	Sensitivity	Specificity	Precision	Recall	F1	Kappa	AUC
5	0.9042	0.8506	0.9453	0.9225	0.8506	0.8851	0.8032	0.9393
10	0.9042	0.8442	**0.9502**	**0.9286**	0.8442	0.8844	0.8029	0.9366
15	0.8986	0.8442	0.9403	0.9155	0.8442	0.8784	0.7917	0.9241
20	**0.9070**	0.8571	0.9453	0.9231	0.8571	**0.8889**	**0.8092**	0.9341
30	0.8901	0.8377	0.9303	0.9021	0.8377	0.8687	0.7745	0.8959
40	0.9042	**0.8636**	0.9353	0.9110	**0.8636**	0.8867	0.8038	**0.9398**
50	0.8761	0.8182	0.9204	0.8873	0.8182	0.8514	0.7454	0.9100
60	0.8873	0.8312	0.9303	0.9014	0.8312	0.8649	0.7685	0.9190
70	0.9042	0.8506	0.9453	0.9225	0.8506	0.8851	0.8032	0.9295
80	0.8873	0.8442	0.9204	0.8904	0.8442	0.8667	0.7692	0.9133
90	**0.9070**	0.8571	0.9453	0.9231	0.8571	**0.8889**	**0.8092**	0.9313
100	0.8845	0.8506	0.9104	0.8792	0.8506	0.8647	0.7640	0.9296

Best-performing model shown in bold.
