# Supplementary material for: Inferring Drug–Gene Relationships in Cancer Using Literature-Augmented Large Language Models
Source: Cancer Res Commun. 2025 Apr 28;5(4):706–18. doi: 10.1158/2767-9764.CRC-25-0030 (PMC12036822; doi:10.1158/2767-9764.CRC-25-0030)
Supplement: Table S3 — Supplementary Table S3 [file crc-25-0030_table_s3_suppst3.pdf]

Supplementary Table S3. Evaluation of various numbers of retrieved abstracts for model performance

Number of abstracts	Accuracy	Sensitivity	Specificity	Precision	Recall	F1	Kappa	AUC
1	0.8697	0.7727	**0.9447**	**0.9154**	0.7727	0.8380	0.7303	0.8755
5	0.8616	0.8636	0.8600	0.8261	0.8636	0.8444	0.7199	0.9054
10	**0.8873**	0.8701	0.9005	0.8701	0.8701	**0.8701**	**0.7706**	**0.9097**
15	0.8701	0.8701	0.8700	0.8375	0.8701	0.8535	0.7368	0.9041
20	0.8305	0.8636	0.8050	0.7733	0.8636	0.8160	0.6598	0.8949
25	0.8559	**0.8896**	0.8300	0.8012	**0.8896**	0.8431	0.7106	0.9019
30	0.8418	0.8831	0.8100	0.7816	0.8831	0.8293	0.6829	0.9019
35	0.8333	0.8571	0.8150	0.7811	0.8571	0.8173	0.6647	0.8787
40	0.8333	0.8636	0.8100	0.7778	0.8636	0.8185	0.6652	0.8792

Best-performing model shown in bold.
