# Supplementary material for: Inferring Drug–Gene Relationships in Cancer Using Literature-Augmented Large Language Models
Source: Cancer Res Commun. 2025 Apr 28;5(4):706–18. doi: 10.1158/2767-9764.CRC-25-0030 (PMC12036822; doi:10.1158/2767-9764.CRC-25-0030)
Supplement: Table S4 — Supplementary Table S4 [file crc-25-0030_table_s4_suppst4.pdf]

Supplementary Table S4. Performance comparison across various LLMs in sentence-level retrieval

Model	Accuracy	Sensitivity	Specificity	Precision	Recall	F1	Kappa	AUC
GPT-4o	0.9042	0.8636	0.9353	0.9110	0.8636	0.8867	0.8038	**0.9398**
Gemini	**0.9211**	**0.8831**	**0.9502**	**0.9315**	**0.8831**	**0.9067**	**0.8385**	0.9228
Llama-3	0.8310	0.8701	0.8010	0.7701	0.8701	0.8171	0.6611	0.8429
Llama-3.2-PubMed	0.7831	0.6039	0.9204	0.8532	0.6039	0.7072	0.5428	0.8294
Mixtral	0.8254	0.7532	0.8806	0.8286	0.7532	0.7891	0.6407	0.8567
Mistral	0.7577	0.8117	0.7164	0.6868	0.8117	0.7440	0.5171	0.7849
BioBERT (sentence)[a]	0.5177	0.6932	0.3985	0.4416	0.6932	0.5365	0.0850	0.5453
BioBERT (drug-gene pair)[b]	0.4954	0.9404	0.1931	0.4418	0.9404	0.6011	0.1130	--

Best-performing LLM model shown in bold.

[a]BioBERT performance on retrieved sentences for target/non-target classification, without considering cancer-type context.

[b]BioBERT performance on drug-gene pairs, obtained by aggregating sentence-level results using majority voting for each pair.
