# Supplementary material for: Inferring Drug–Gene Relationships in Cancer Using Literature-Augmented Large Language Models
Source: Cancer Res Commun. 2025 Apr 28;5(4):706–18. doi: 10.1158/2767-9764.CRC-25-0030 (PMC12036822; doi:10.1158/2767-9764.CRC-25-0030)
Supplement: Table S5 — Supplementary Table S5 [file crc-25-0030_table_s5_suppst5.pdf]

Supplementary Table S5. Performance comparison across various LLMs in abstract-level retrieval

Model	Accuracy	Sensitivity	Specificity	Precision	Recall	F1	Kappa	AUC
GPT-4o	0.8873	0.8701	0.9005	0.8701	0.8701	0.8701	0.7706	**0.9097**
Gemini	**0.8986**	0.8052	**0.9701**	**0.9538**	0.8052	**0.8732**	**0.7897**	0.9090
Llama-3	0.8592	0.7792	0.9204	0.8824	0.7792	0.8276	0.7093	0.8703
Llama-3.2-PubMed	0.7099	**0.8955**	0.4675	0.6870	**0.8955**	0.7775	0.3807	0.7668
Mixtral	0.8451	0.7403	0.9254	0.8837	0.7403	0.8057	0.6785	0.8304
Mistral	0.6648	0.8052	0.5572	0.5822	0.8052	0.6757	0.3469	0.7434

Best-performing model shown in bold.
