# Supplementary material for: Inferring Drug–Gene Relationships in Cancer Using Literature-Augmented Large Language Models
Source: Cancer Res Commun. 2025 Apr 28;5(4):706–18. doi: 10.1158/2767-9764.CRC-25-0030 (PMC12036822; doi:10.1158/2767-9764.CRC-25-0030)
Supplement: Table S6 — Supplementary Table S6 [file crc-25-0030_table_s6_suppst6.pdf]

Supplementary Table S6. Performance comparison across various LLMs without retrieval

Model	Accuracy	Sensitivity	Specificity	Precision	Recall	F1	Kappa	AUC
GPT-4o	**0.9174**	0.8158	**0.9950**	**0.9920**	0.8158	**0.8953**	**0.8281**	0.8831
Gemini	0.8986	0.7987	0.9751	0.9609	0.7987	0.8723	0.7894	0.8736
Llama-3	0.8676	0.7662	0.9453	0.9147	0.7662	0.8339	0.7253	0.7971
Llama-3.2-PubMed	0.7690	0.8442	0.7114	0.6915	0.8442	0.7602	0.5416	0.8205
Mixtral	0.8901	0.7532	0.9950	0.9915	0.7532	0.8561	0.7699	0.8016
Mistral	0.8704	**0.8701**	0.8706	0.8375	**0.8701**	0.8535	0.7374	**0.9059**

Best-performing model shown in bold.
