# Supplementary material for: Inferring Drug–Gene Relationships in Cancer Using Literature-Augmented Large Language Models
Source: Cancer Res Commun. 2025 Apr 28;5(4):706–18. doi: 10.1158/2767-9764.CRC-25-0030 (PMC12036822; doi:10.1158/2767-9764.CRC-25-0030)
Supplement: Table S8 — Supplementary Table S8 [file crc-25-0030_table_s8_suppst8.pdf]

Supplementary Table S8. Top hub genes in the pan-cancer drug-gene interaction network

Gene	Degree
MTOR	45
BCL2	41
STAT3	40
EGFR	31
AR	29
MYC	25
JUN	20
SRC	18
MYCN	17
FLT3	14
JAK2	14
ALK	14
MDM2	13
KIT	13
CDK4	12
MET	12
BRAF	10
CCND1	10
FLI1	10
ERBB2	10
ROS1	10
FGFR1	10
RET	10
NRAS	10
CXCR4	10

Degree represents the number of genes interacting with each drug in the network.
