# Supplementary material for: Inferring Drug–Gene Relationships in Cancer Using Literature-Augmented Large Language Models
Source: Cancer Res Commun. 2025 Apr 28;5(4):706–18. doi: 10.1158/2767-9764.CRC-25-0030 (PMC12036822; doi:10.1158/2767-9764.CRC-25-0030)
Supplement: Table S9 — Supplementary Table S9 [file crc-25-0030_table_s9_suppst9.pdf]

Supplementary Table S9. Top hub drugs in the pan-cancer drug-gene interaction network

Gene	Degree
Cisplatin	44
Sorafenib	33
Regorafenib	27
Paclitaxel	25
Tamoxifen	24
Gemcitabine	22
Temozolomide	22
Trametinib	22
Crizotinib	20
Cabozantinib	18
Doxorubicin	18
Lenvatinib	18
Sunitinib	18
Everolimus	16
5-fluorouracil	15
Axitinib	14
Gefitinib	14
Palbociclib	14
Pazopanib	14
Imatinib	13
Olaparib	13

Degree represents the number of genes interacting with each drug in the network.
