# Supplementary material for: Inferring Drug–Gene Relationships in Cancer Using Literature-Augmented Large Language Models
Source: Cancer Res Commun. 2025 Apr 28;5(4):706–18. doi: 10.1158/2767-9764.CRC-25-0030 (PMC12036822; doi:10.1158/2767-9764.CRC-25-0030)
Supplement: Figure S1 — Supplementary Figure S1 [file crc-25-0030_figure_s1_suppsf1.pdf]

Context

You are a biomedical AI system analyzing the relationship between a specific gene and a specific drug in a particular type of cancer. Focusing on the sentences provided, you will:

Chain-of-thought Inference

1. Summarize the key points from all the sentences.

2. Make an inference about the targeting relationship between the drug and the gene in the specified cancer based on the sentences, using this format:

The drug <drug name> <appears to target, does not appear to target, or is undetermined for> the gene <gene name> in <cancer type> with <high, medium, or low> confidence.

3. Identify the mechanism of targeting relationship. If the relationship is inferred as ‘appears to target’, specify the following targeting mechanism <Direct binding or Indirect modulation>. If the relationship is inferred as ‘does not appear to target, or is undetermined for’, indicate as <NA>.

4. Identify and summarize the major cancer type associated with the gene-drug relationship from all relevant sentences (e.g., liver cancer, lung cancer, breast cancer). Use broad categories and avoid specific subtypes unless necessary. Use commas (,) to separate if including more than one cancer type.

5. Provide step-by-step explanations for your inference, using full sentences or paragraphs without numeric list indicators like 1., 2., or newline characters ('\n'). If there is ambiguous or contradictory information within the explanation, describe how these issues were resolved during the analysis.

Definitions

Note: A 'targeting relationship' exists if the drug specifically targets the gene in the cancer. We define two targeting mechanisms: 'Direct binding' and 'Indirect modulation.' A 'Direct binding' mechanism means the drug directly binds to a specific protein encoded by a gene, influencing its activity to produce the intended therapeutic effect. An 'Indirect modulation' mechanism refers to how genetic variations can modify drug response and how drugs can influence cellular behavior by regulating gene expression. If evidence is insufficient, the relationship should be classified as 'undetermined.' Please ensure the identified major cancer types match the inference of the relationships between gene-drug and cancer.

Output Format

Please present your response in JSON format, adhering to this structure:

{\"Summary\":<value>, \"Inference\":<value>, \"Explanation\":<value>, \"Mechanism\":<value>,
\"Major cancer type\":<value>}

Note: Be concise and avoid overflow in each section.

Retrieved Information

Gene: {Gene from Query}
Drug: {Drug from Query}

Sentences to be used:
{
Sentence1
Sentence2
...
Sentence40
}

Supplementary Fig. S1. Alternative prompt design. The prompt is designed for constructing the drug-gene interaction network that covers both direct binding and indirect modulation relationships and identifies the major cancer types. The primary prompt, designed solely for direct relationships and performance evaluation, is shown in Fig. 1B of the main text.
