# Supplementary material for: Inferring Drug–Gene Relationships in Cancer Using Literature-Augmented Large Language Models
Source: Cancer Res Commun. 2025 Apr 28;5(4):706–18. doi: 10.1158/2767-9764.CRC-25-0030 (PMC12036822; doi:10.1158/2767-9764.CRC-25-0030)
Supplement: Figure S2 — Supplementary Figure S2 [file crc-25-0030_figure_s2_suppsf2.pdf]

Inference	Not target			Undetermined			Target		
Confidence	High	Medium	Low	Low	Medium	High	Low	Medium	High
Score	1	2	3	4	5	6	7	8	9

Supplementary Fig. S2. Scoring system for quantifying LLM inferences for performance evaluation. The scoring system ranges from 1 to 9 and is based on both the inferred association type (not target, undetermined, target) and the confidence level (low, medium, high). Each inference by the LLM is accompanied by a confidence level, with "low" indicating less certainty and "high" indicating greater certainty. The scores are used for systematic performance evaluation.
