# Supplementary material for: Inferring Drug–Gene Relationships in Cancer Using Literature-Augmented Large Language Models
Source: Cancer Res Commun. 2025 Apr 28;5(4):706–18. doi: 10.1158/2767-9764.CRC-25-0030 (PMC12036822; doi:10.1158/2767-9764.CRC-25-0030)
Supplement: Figure S3 — Supplementary Figure S3 [file crc-25-0030_figure_s3_suppsf3.pdf]

Query: Metformin, BRCA2, Breast Cancer
Model: GPT-4o with <u>sentence-level retrieval</u>

Inference

The drug Metformin **does not appear to target** the gene *BRCA2* in breast cancer **with high confidence**.

Summary

The sentences emphasize that *BRCA2* is a significant target in breast cancer, particularly in the context of targeted therapies like *PARP* inhibitors. *BRCA2* mutations are associated with homologous recombination DNA repair defects, making them susceptible to specific treatments. Additionally, the role of *BRCA2* in DNA repair and its interaction with other genes and proteins like *RAD51*, *FoxM1*, and *XRCC1* are highlighted. Other potential targets and therapies, such as *mTORC1* and *PI3KCA*, are also mentioned in the context of *BRCA2*.

Explanation

The provided sentences focus on the treatment of *BRCA2*-mutated breast cancers using various targeted therapies, particularly *PARP* inhibitors. Other targets and genes related to *BRCA2* in breast cancer, such as *mTORC1* and *PI3KCA*, are also discussed. However, there is no mention of Metformin being used to target *BRCA2* in breast cancer specifically. The consistent absence of Metformin in the context of *BRCA2* and breast cancer across multiple sentences leads to a high confidence conclusion that Metformin does not directly target *BRCA2* in this cancer type.

Supplementary Fig. S3. Output of our pipeline for a negative drug-gene pair (metformin and *BRCA2* in breast cancer).
