# Supplementary material for: Inferring Drug–Gene Relationships in Cancer Using Literature-Augmented Large Language Models
Source: Cancer Res Commun. 2025 Apr 28;5(4):706–18. doi: 10.1158/2767-9764.CRC-25-0030 (PMC12036822; doi:10.1158/2767-9764.CRC-25-0030)
Supplement: Figure S4 — Supplementary Figure S4 [file crc-25-0030_figure_s4_suppsf4.pdf]

Query: CX-5461, TOP2B, Cancer
Model: GPT-4o <u>without retrieval</u>

Inference

The drug CX-5461 **does not appear to target** the gene *TOP2B* in cancer **with high confidence**.

Explanation

CX-5461 is primarily known as a **selective inhibitor of RNA polymerase I transcription** and has been shown to target the ribosomal RNA (rRNA) synthesis pathway. Its anticancer activity is largely attributed to its ability to inhibit rRNA synthesis, leading to nucleolar stress and activation of the p53 pathway, which induces apoptosis in cancer cells. On the other hand, *TOP2B* encodes topoisomerase II beta, an enzyme involved in DNA replication, transcription, and cell cycle control, which is different from the pathways influenced by CX-5461. There is **no substantial evidence or literature** indicating that CX-5461 directly targets *TOP2B*. Therefore, based on the distinct mechanisms of action of CX-5461 and the lack of specific evidence linking it to *TOP2B*, it can be inferred with high confidence that CX-5461 does not directly target *TOP2B* in cancer.

Supplementary Fig. S4. Output of our pipeline for CX-5461, a drug with a historically misclassified target, without retrieved information. CX-5461 was long thought to target RNA Polymerase I until evidence from 2021 identified *TOP2B* as the primary target. Without retrieval, GPT-4o (version 2024-05-13) fails to recognize *TOP2B* as the target, likely due to outdated information.
