# Supplementary material for: Inferring Drug–Gene Relationships in Cancer Using Literature-Augmented Large Language Models
Source: Cancer Res Commun. 2025 Apr 28;5(4):706–18. doi: 10.1158/2767-9764.CRC-25-0030 (PMC12036822; doi:10.1158/2767-9764.CRC-25-0030)
Supplement: Figure S5 — Supplementary Figure S5 [file crc-25-0030_figure_s5_suppsf5.pdf]

Query: *OTS964, CDK11, Cancer*
Model: GPT-4o <u>without retrieval</u>

Inference

The drug OTS964 **does not appear to target** the gene *CDK11* in cancer **with high confidence**.

Explanation

OTS964 is a known potent inhibitor of the protein kinase *TOPK* (T-lymphokine-activated killer cell-originated protein kinase), which is implicated in various cancers. **The available scientific literature and drug databases do not indicate that OTS964 directly targets *CDK11*.** *CDK11*, or Cyclin-Dependent Kinase 11, is another kinase that plays a role in cell cycle regulation, but no direct targeting relationship between *CDK11* and OTS964 has been established. Given the specificity of OTS964 for *TOPK* and the lack of evidence linking it to *CDK11*, the inference is made with high confidence that OTS964 does not target *CDK11* in cancer.

Query: OTS964, CDK11, Cancer
Model: GPT-4o with <u>sentence-level retrieval</u>

Inference

The drug OTS964 **appears to target** the gene *CDK11* in cancer **with high confidence**.

Summary

OTS964 binds to and inhibits the activity of *CDK11*, a cyclin-dependent kinase, in various cancer cell lines. Crystal structures demonstrate that OTS964 occupies the ATP binding pocket of *CDK11*, favoring its active conformation. **OTS964 was initially developed to target another kinase, *PBK*, but was found to have significant off-target effects on *CDK11*, which contribute to its anti-cancer properties.** The specificity of OTS964 for *CDK11* over other CDKs is influenced by the unique xDFG motif of *CDK11*.

Explanation

The evidence provided strongly supports a direct targeting relationship between OTS964 and *CDK11* in cancer. Multiple sentences confirm that OTS964 **binds specifically to** the *CDK11* kinase domain, occupies the ATP binding pocket, and promotes the active conformation of *CDK11*. Additionally, crystal structures and biochemical assays demonstrate the interaction and inhibitory effects of OTS964 on *CDK11*. Although OTS964 was initially developed to target *PBK*, its significant off-target activity on *CDK11* was identified and shown to be responsible for its anti-cancer effects. This consistent and detailed evidence leads to a high-confidence inference that OTS964 targets *CDK11* in cancer.

Supplementary Fig. S5. Output of our pipeline for OTS964 targeting *CDK11* in cancer. Upper panel: the drug OTS964 was initially developed to target PBK but was later found to have a significant off-target effect on *CDK11*, contributing to its anti-cancer properties. Without retrieval, GPT-4o does not recognize *CDK11* as the target, likely due to outdated information. **Lower panel:** Using sentence-level retrieval, our pipeline correctly identified *CDK11* as the specific target of OTS964 in cancer with high confidence.
