# Supplementary material for: Inferring Drug–Gene Relationships in Cancer Using Literature-Augmented Large Language Models
Source: Cancer Res Commun. 2025 Apr 28;5(4):706–18. doi: 10.1158/2767-9764.CRC-25-0030 (PMC12036822; doi:10.1158/2767-9764.CRC-25-0030)
Supplement: Figure S6 — Supplementary Figure S6 [file crc-25-0030_figure_s6_suppsf6.pdf]

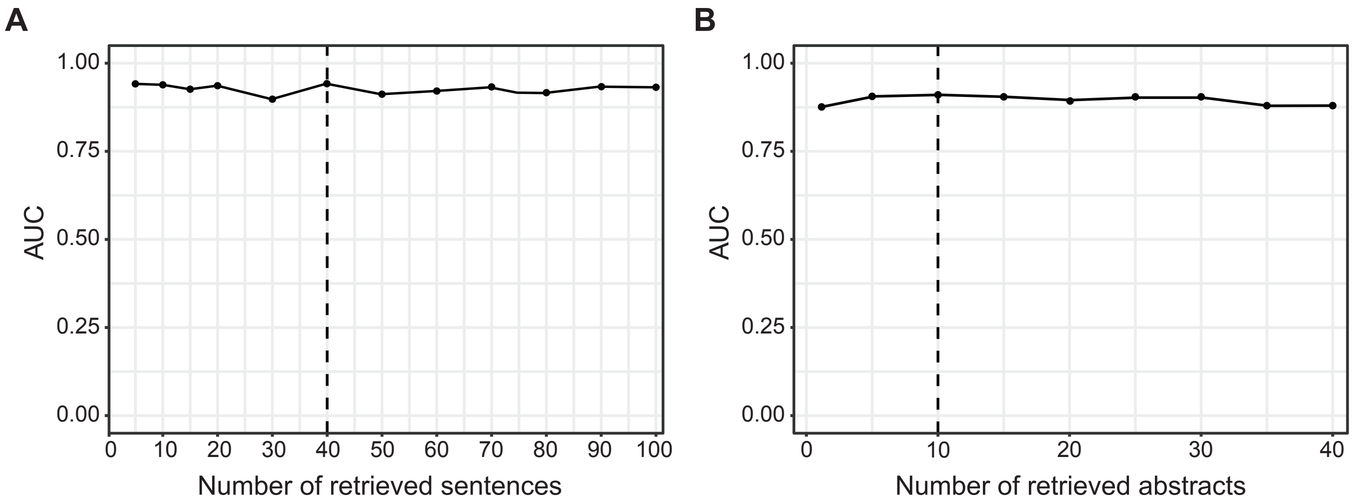

Supplementary Fig. S6. Performance evaluation of retrieval approaches. Area under the receiver operating characteristic curve (AUC) across varying numbers of **(A)** retrieved sentences and **(B)** abstracts used for the inference. The AUC measures the ability of the LLM inference method to distinguish between positive and negative drug-gene cases curated from two large databases, Cancer Drugs DB and PharmGKB. Dashed lines indicate the optimal numbers of retrieved sentences and abstracts selected for follow-up analysis. Detailed results are available in Supplementary Tables S2-S3.
