# Supplementary material for: Inferring Drug–Gene Relationships in Cancer Using Literature-Augmented Large Language Models
Source: Cancer Res Commun. 2025 Apr 28;5(4):706–18. doi: 10.1158/2767-9764.CRC-25-0030 (PMC12036822; doi:10.1158/2767-9764.CRC-25-0030)
Supplement: Figure S7 — Supplementary Figure S7 [file crc-25-0030_figure_s7_suppsf7.pdf]

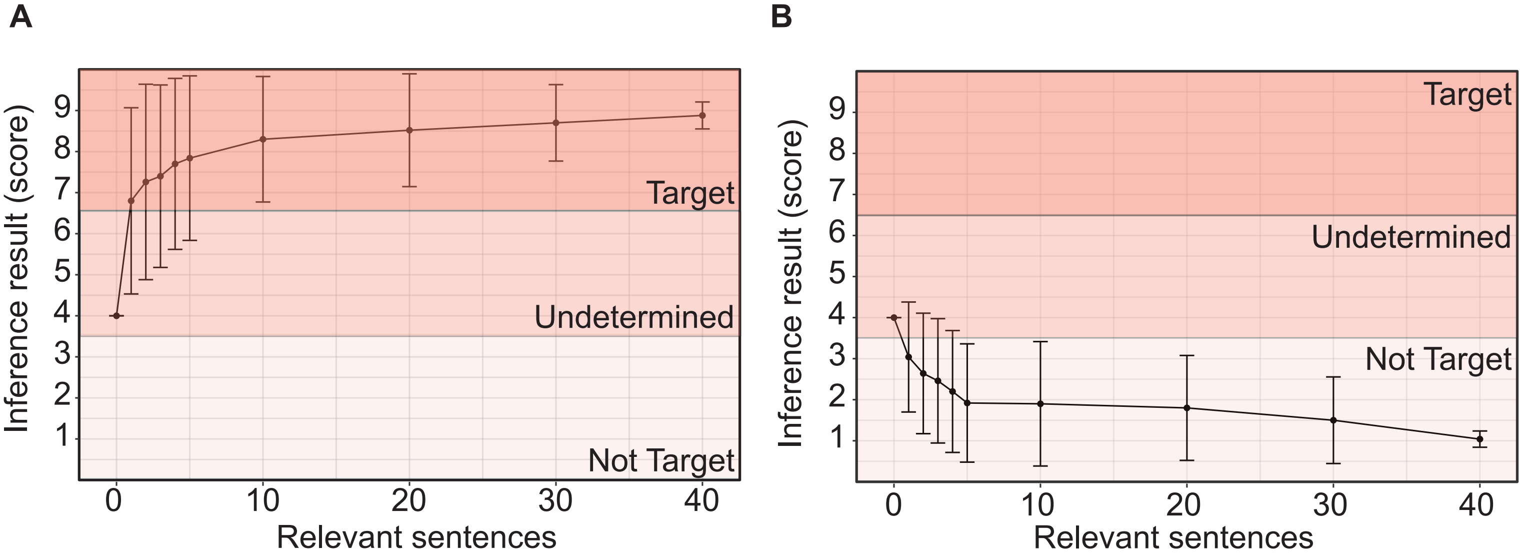

Supplementary Fig. S7. Evaluation of confidence levels by a contamination test. (A) Results of 50 randomly selected positive drug-gene pairs in corresponding cancer types. The contamination test assesses how LLM inference is affected by the mixture of relevant and irrelevant sentences included in the retrieved information. The resulting score shows an upward trend from 4 (undetermined with low confidence) to 9 (target with high confidence) as the number of relevant sentences increases, indicating increased confidence in the output. **(B)** Results of 50 randomly selected negative drug-gene pairs. The resulting score shows a downward trend from 4 (undetermined with low confidence) to 1 (not target with high confidence) as the number of relevant sentences increases, similarly indicating increased confidence in the output.
